# Multivariate Analysis on the Properties of Intact Cereal Kernels and Their Association with Viscoelasticity at Different Moisture Contents

**DOI:** 10.3390/foods12040808

**Published:** 2023-02-14

**Authors:** Anayansi Escalante-Aburto, Juan de Dios Figueroa-Cárdenas, Aurelio Dominguez-Lopez, Silverio García-Lara, Néstor Ponce-García

**Affiliations:** 1 Tecnologico de Monterrey, The Institute for Obesity Research, Monterrey 64700, Mexico; 2 Centro de Investigación y Estudios Avanzados (CINVESTAV) Unidad Querétaro, Querétaro 76230, Mexico; 3 Facultad de Ciencias Agrícolas, Universidad Autónoma del Estado de Mexico (UAEMex), Toluca 50200, Mexico; 4 Tecnologico de Monterrey, Escuela de Ingeniería y Ciencias, Monterrey 64700, Mexico

**Keywords:** cereals, biophysical properties, uniaxial compression, viscoelasticity, multivariate analysis

## Abstract

The viscoelastic properties of cereal kernels are strongly related to their quality, which can be applied to the development of a more selective and objective classification process. In this study, the association between the biophysical and viscoelastic properties of wheat, rye, and triticale kernels was investigated at different moisture contents (12% and 16%). A uniaxial compression test was performed under a small strain (5%), and the increase in viscoelasticity at 16% moisture content corresponded to proportional increases in biophysical properties such as the appearance and geometry. The biophysical and viscoelastic behaviors of triticale were between those of wheat and rye. A multivariate analysis showed that the appearance and geometric properties significantly influenced kernel features. The maximum force showed strong correlations with all viscoelastic properties, and it can be used to distinguish between cereal types and moisture contents. A principal component analysis was performed to discriminate the effect of the moisture content on different types of cereals and to evaluate the biophysical and viscoelastic properties. The uniaxial compression test under a small strain and the multivariate analysis can be considered a simple and non-destructive tool for assessing the quality of intact cereal kernels.

## 1. Introduction

Cereals are considered the primary nutrient source of the global population, especially in emergent economies. The main cereals cultivated globally are maize, rice, and wheat, which account for 90% of total production [1]. However, sustainability issues have challenged traditional agricultural production and consumption practices. Significant losses of cereals and other food commodities occur because of poor kernel quality and handling during operations such as harvesting, transportation, storage, conditioning, and milling [2]. The commercial practice of conditioning wheat before milling alters the mechanical properties of the kernels and improves the milling properties. After wheat, rye is the second-most widely used cereal for bread production [3], and most of its production is intended for grinding to produce flour [4]. Meanwhile, triticale is an artificially created cross between wheat and rye, so its morphological and biological attributes are usually between those of its progenitors [5]. Despite good crop yields, triticale has a low milling yield, and it is mainly used as an alternative to wheat [6]. Both rye and triticale are usually incorporated into wheat flour to enrich or improve the nutritional quality of the product [7]. Studying their biophysical, mechanical, and viscoelastic properties is important to improve their viability as an alternative ingredient and their behavior during processing.

Substantial research efforts have been dedicated to establishing the relationship between the protein composition and viscoelastic properties of doughs for improved bread-making quality. However, very little work has been dedicated to examining the relationship between the biophysical and viscoelastic properties of intact wheat kernels [2]. Ponce-García et al. [8] reported a simple and practical method for evaluating the fundamental mechanical properties of intact wheat kernels without any preparation to increase the selection efficiency for processing, marketing, and end-use. The size, shape, weight, density, and volume of kernels used as the raw material for processing have a significant relationship with several technological requirements. The shape and size of kernels are closely related to their mechanical and viscoelastic properties. The viscoelasticity of kernels can be used as a practical approach to recognize and predict the potential use and industrial performance of different cereal types [9,10]. However, the mechanical properties of conditioned wheat kernels have not been investigated sufficiently, let alone those of rye and triticale kernels, despite the critical technological implications.

To the best of the authors’ knowledge, the rheological features of rye and triticale have been studied in flours but not in whole kernels. Multivariate analysis is a useful tool for evaluating the various characteristics of cereal kernels and flours to predict their agronomic and technological properties. Wikström and Bohlin [11] evaluated the mixogram parameters of 21 wheat cereal types to predict their baking quality, such as bread volume. Ali et al. [12] used multivariate analysis to assess 64 advanced wheat lines for breeding programs. Lima et al. [13] used multivariate analysis to study the effects of drying and storage conditions on the physicochemical quality of kernels. They concluded that early harvest of soybeans reduced losses in the field and increased the flow in silos.

The moisture content is a critical parameter for evaluating whole kernels because pre- and post-harvesting operations such as storage and milling require certain levels of moisture for optimal processing [14]. The moisture content of rye and triticale kernels can be conditioned to improve their viscoelastic behavior and flour yield, which would be useful for increasing their utility and value. The objective of this study was to characterize and evaluate the biophysical, geometric, mechanical, and viscoelastic properties of wheat, rye, and triticale kernels at two moisture levels. Univariate and multivariate analyses were performed on the obtained data to develop a prediction model of the kernel quality for the different cereals according to the moisture content.

## 2. Materials and Methods

### 2.1. Biological Materials

Three cereal types were used in this study: wheat (*Triticum aestivum* L. var. *Rayón*), rye (*Secale cereale* L. var. *Criollo Tlaxcala*), and triticale (X *Triticosecale* Wittmack, var. *Rebeca*). Twenty samples were collected for each cereal type. All samples were acquired in 2021 from a local seed supplier in Toluca, Estado de Mexico. The wheat cultivar is a hard variety and usually is cultivated in the summer–fall season, under irrigation conditions. With respect to rye, this variety is only cultivated in Mexico in rainfed conditions and mild climates. Triticale kernels are cultivated in the highlands due to their tolerance to low temperatures and drought conditions. Samples were cleaned of foreign materials by using an aluminum sieve with triangular perforations with side lengths of 1.98 mm (5/64″, Seedburo Equipment Co., Des Plaines, IL, USA). Each sample was then placed in a separate polyethylene bag, hermetically sealed, and stored at 4 °C until use.

### 2.2. Moisture Content and Conditioning of Kernels

The moisture content for each cereal type was assessed before and after kernel conditioning [15]. For each sample, 250 g of kernels were conditioned at different moisture contents (12% and 16%, *w/w*) following the method proposed by Serna [16]. The samples were then stored in sealed polyethylene bags at 4 °C until use. These moisture contents were selected by considering the optimal storage and milling conditions used by industrial processors.

### 2.3. Evaluation of Biophysical, Geometric, and Mechanical Properties of Intact Kernels

Fourteen properties were evaluated and grouped into four categories: appearance (two), geometric (four), weight/volume/density (six), and mechanical (two) [17]. Twenty kernels from each sample were randomly selected for each cereal type at two moisture contents (12% and 16%, *w*/*w*). In other words, 20 samples with each comprising 20 kernels were obtained at the different moisture contents. This resulted in 400 kernels at 12% moisture content and 400 kernels at 16% moisture content, or 800 kernels in total for each cereal type (see Supplementary Material).

#### 2.3.1. Appearance

The length (*L*) and width (*W*) were measured in millimeters by using a digital caliper (Model CD-6 CS, Mitutoyo, Japan). The thickness (*T*) was calculated by using a texture analyzer (TA-XT2 Plus, Texture Technologies Corporation, Stable Micro Systems, Surrey, England) according to the method used by Ponce-García et al. [18]. An adjustment was applied to reach 5% deformation:

(1) $$T=\left(\frac{d}{5}\right)\times \;100$$

where *d* is the displacement due to load.

The width/length ratio (*R_WL_*) was calculated as follows by using the method of Mohsenin [17] and Markowski et al. [19]:

(2) $$R_{WL}=\left(\frac{W}{L}\right)\times \;100$$

#### 2.3.2. Geometric Properties

The geometric diameter (*D_g_*) and arithmetic diameter (*D_a_*) were calculated as follows [20].

(3) $$D_{g}={\left(LWT\right)}^{1/3}$$

(4) $$\;D_{a}=\frac{\left(LWT\right)}{3}$$

The kernel surface area (*SA*) was calculated as follows [20]:

(5) $$SA=\pi \;{\left(D_{g}\right)}^{2}$$

The sphericity (Φ) was calculated as a percentage [17,21]:

(6) $$\Phi =[\frac{D_{g}}{L}]x100$$

#### 2.3.3. Weight, Volume, and Density

The individual kernel weight (*IKW*) was obtained by weighing 20 samples separately (g) on an analytical scale (OHAUS, model BBL61, Boeco, Hamburg, Germany). The thousand kernel weight (*TKW*) was obtained according to the procedure described by Ponce-García et al. [22]. The ellipsoidal volume ($E_{v}$) was calculated by using the axial dimensions of the kernels according to Markowski et al. [19]:

(7) $$E_{v}=\frac{\pi }{6}\cdot \left(LWT\right)$$

The apparent density in bulk ($\rho_{b}$) was calculated according to the 55–10.01 method [23] and Bhises et al. [24]:

(8) $$\rho_{b}=\frac{w_{s}}{V_{s}}$$

The real density ($\rho_{t}$) was calculated as follows [25]:

(9) $$\rho_{t}=\frac{P_{g}}{V_{d}}$$

where *P_g_* is the kernel weight (g) and *V_d_* is the displacement volume (mL).

The porosity ($\varepsilon_{v}$) was calculated as follows [17]:

(10) $$\varepsilon_{v}=\left(1-\frac{\rho_{b}}{\rho_{t}}\right)\times \;100$$

#### 2.3.4. Mechanical Properties

The maximum force at 5% deformation (*F_max_*) and the hardness (*H*) were calculated from the measurement data of a texture analyzer (TA-XT2 Plus) equipped with a probe SMSP/2 (2 mm) and following the procedure of Ponce-García et al. [18].

#### 2.3.5. Viscoelastic Performance

The viscoelastic performance was assessed by using measurement data from a texture analyzer TA-XT2 Plus with a probe model SMS P/1KS (10 mm). The method of uniaxial compression at small deformation reported by Ponce-García et al. [22] was used with two modifications. First, the simple compression mode was substituted by a TPA mode (i.e., double compression). Second, the deformation index during compression was increased from 3% to 5%. The pretest, test, and post-test velocities were set to 1, 0.1, and 0.1 mm s^−1^, respectively. Twenty kernels from each sample were randomly selected for each cereal type at the two moisture contents (12% and 16%, *w/w*). The elastic modulus (*E*) was obtained at 5% deformation [8]. The total work (*W_t_*), elastic work (*W_e_*), and plastic work (*W_p_*) were calculated from the load–unload curves obtained after compression from the texture analyzer based on the methods proposed by Ponce-García et al. [8] and Gubicza et al. [26]. The degree of elasticity (*D_e_*) and resilience (*R*) were calculated according to the method of Figueroa et al. [2].

### 2.4. Design of Experiments and Statistical Analysis

A completely randomized trial was used. A factorial design with two factors was used: the cereal type (three levels) and moisture content (two levels). The average values were calculated from 20 samples for each cereal type. An analysis of variance was performed at a significance level of 95%, and a multiple-media comparison was performed to evaluate differences among treatments by the Tukey test (*p* < 0.05) using the statistical software SAS^®^ [27]. Data were evaluated in the univariate and multivariate directions (*p* < 0.05) via Pearson’s linear correlation analysis and principal component analysis (PCA), respectively, using the software PAST^®^ [28].

## 3. Results

### 3.1. Effect of Moisture Content on the Biophysical Properties

**Figure 1:**
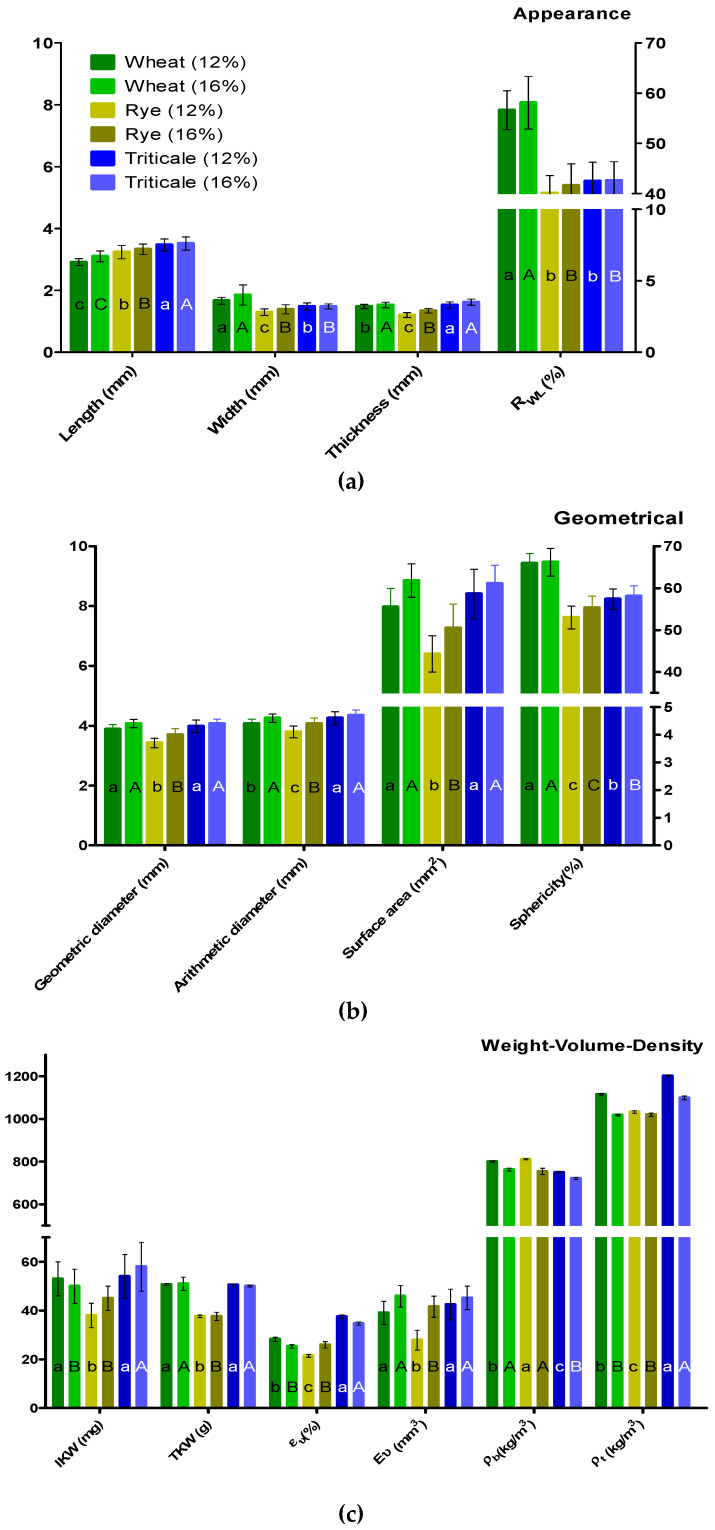
Mean ± standard deviation for the appearance (**a**), geometric (**b**), and weight/volume/density properties (**c**) of the three cereal types at two moisture contents (12% and 16%). *R_LW_*: length/width ratio; *IKW*: individual kernel weight; *TKW*: thousand kernel weight; $\varepsilon_{v}$: porosity in bulk; *E_υ_*: ellipsoidal volume; $\rho_{b}$: apparent density; $\rho_{t}$: true density. Mean values for the same cereal type with different letters (lowercase and uppercase) were statistically different (*p* < 0.05) at the different moisture contents (12% and 16%, respectively).

Figure 1 shows the biophysical properties of the three cereal types conditioned at two different moisture contents. Significant differences were observed among the cereal types in the appearance properties. This can be attributed to the large natural variability in the cereal spikes, which is a product of their natural morphology. The three cereal types differed in all appearance properties, which increased as a function of the moisture content. The geometric properties indicated that the moisture content had a significant effect within the same cereal type and among different cereal types. The three cereal types generally showed significant increases in these properties with increasing moisture content. However, wheat showed no differences in *D_g_* and *SA* at different moisture contents, and triticale showed no differences in *D_g_*, *D_a_*, and *SA*. In contrast, rye kernels showed differences in almost all geometric properties with different moisture contents, which can be attributed to its genomic origin. In general, significant differences were observed within and among cereal types at different moisture contents for the weight/volume/density properties. In particular, both *ρ_b_* and *ρ_t_* decreased with increasing moisture content for the three cereal types. As expected, the *TKW* and *ρ_b_* values were similar to those of many kernel-grading systems worldwide. However, the *SA* and *TKW* values for triticale were higher than reported elsewhere. In this study, *ε_v_* showed significant differences depending on the cereal type and moisture content. Furthermore, the moisture content had significant effects on geometric properties such as Φ and $E_{v}$, as well as properties related to the kernel weight to a lesser extent.

### 3.2. Effect of Moisture Content on the Mechanical Properties and Viscoelastic Performance

**Figure 2:**
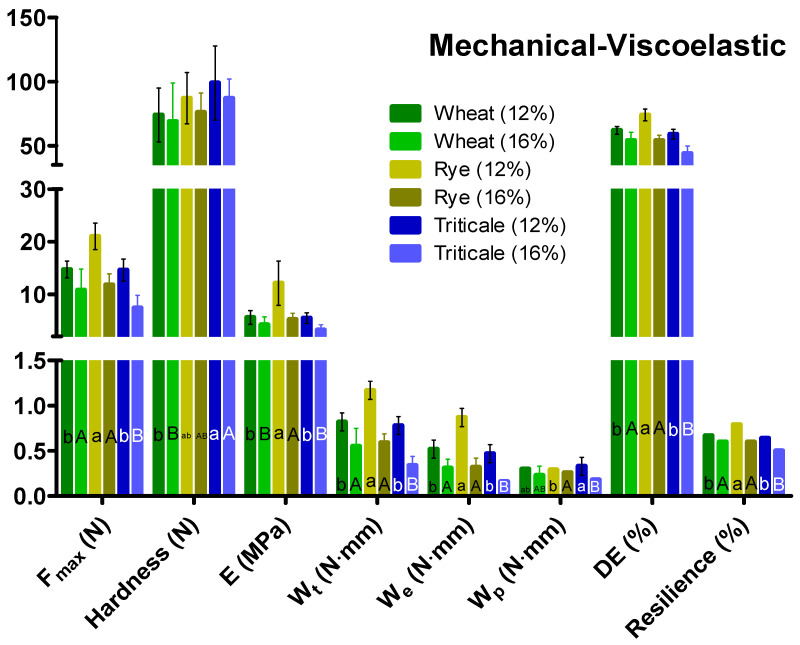
Mean ± standard deviation of the mechanical properties and viscoelastic performance of wheat, rye, and triticale kernels at different moisture contents. *F_max_*: maximum force; *E*: elastic modulus; *W_t_*: total work; *W_e_*: elastic work; *W_p_*: plastic work; *DE*: degree of elasticity. Mean values for the same cereal type with different letters (lowercase and uppercase) were statistically different (*p* < 0.05) at the different moisture contents (12% and 16%, respectively).

**Figure 3:**
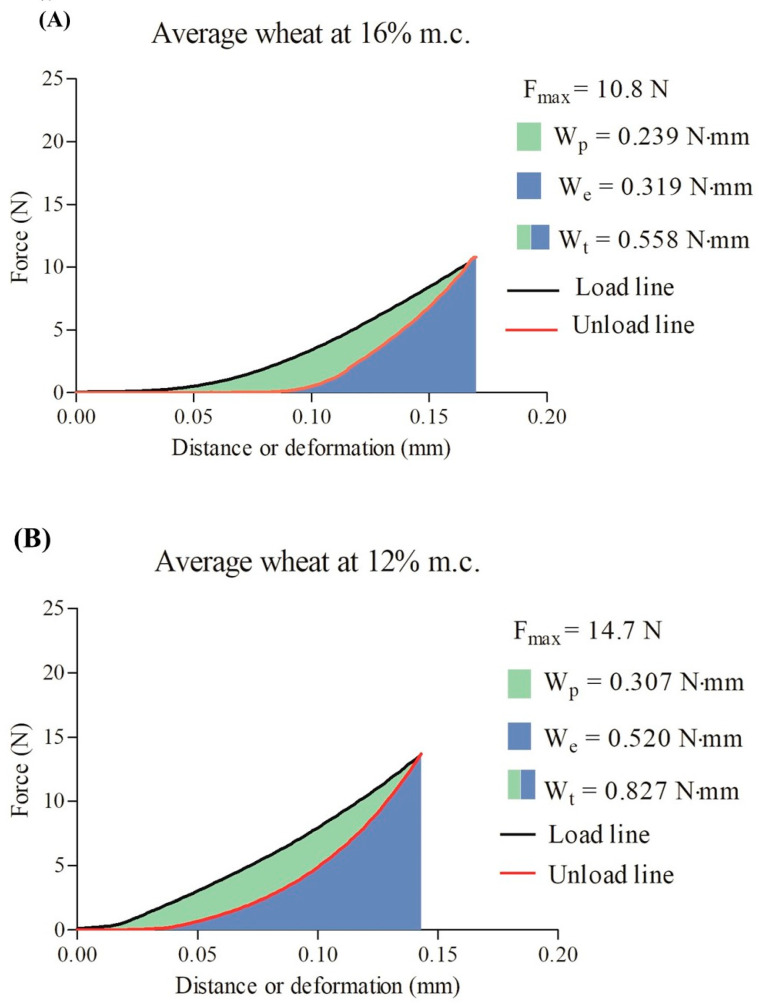
Force–deformation curves obtained from the uniaxial compression test at small deformation (5%) of the kernels at different moisture contents: (**A**) 16% and (**B**) 12%. m.c.: moisture content; *F_max_*: maximum force; *W_p_*: plastic work; *W_e_*: elastic work; *W_t_*: total work.

Figure 2 depicts the mechanical properties and viscoelastic performance of the cereals at the two moisture contents. Rye showed a higher *F_max_* and *H* than wheat and triticale at both moisture contents. Conditioning decreased *F_max_* and *H* by about 39.8% and 31.4%, respectively, for the three cereal types. However, wheat was less affected than rye and triticale. Increasing the moisture content significantly decreased the mechanical properties and viscoelastic performance, which can affect industrial operations such as milling, as indicated by the significant differences in *W_t_*. Significant differences were observed within and among the cereals in terms of *E*, *W_t_*, *W_e_*, *W_p_*, *D_e_*, and *R*. Rye had a very high *E* and *W_t_*, but conditioning decreased *E* and *W_t_* to values similar to those for wheat and triticale. Wheat and triticale showed similar viscoelastic behavior for all properties at a moisture content of 12%; however, the values of the properties decreased when the moisture content was increased to 16%. As expected, conditioning decreased *W_t_* by about 32.9%, 49.5%, and 56.4% for wheat, rye, and triticale, respectively. Figure 3 shows an example of the force–deformation curves obtained during the uniaxial compression test. Conditioning kernels at a higher moisture content decreased *F_max_* and affected the differences in *W_e_*, *W_p_*, and *W_t_*.

### 3.3. Multivariate Analysis

**Figure 4:**
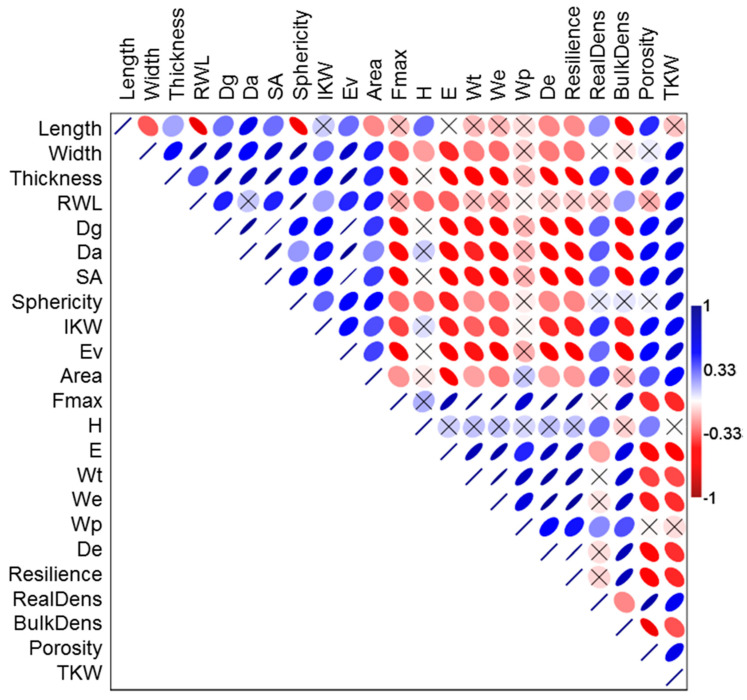
Correlation matrix among the variables of the biophysical and viscoelastic properties of the three cereal types conditioned at two moisture contents (12% and 16%). The crossed ellipses indicate no significant correlation at *p* > 0.05 (independent of color), and the slope and color define the correlation between each pair of variables (positive or negative).

Pearson’s correlation analysis was performed for an initial identification of variables with the most important and significant associations. Figure 4 depicts the correlations among the variables encompassing the biophysical and viscoelastic properties. *L* had strongly negative correlations with *R_WL_*, *Φ*, and *ρ_b_*. In contrast, *W* had positive correlations with *R_WL_* and most geometric properties. *T* had positive correlations with all of the geometric properties and densities. The geometric properties had negative correlations with the viscoelastic properties except for *W_p_* and $\rho_{b}$. *F_max_* is widely used to calculate the viscoelastic and rheological properties of a kernel. *F*_max_ showed strongly positive correlations with all of the viscoelastic properties and $\rho_{b}$. Finally, *TKW* showed negative correlations with most of the appearance, geometric, weight, and volume properties. This is significant because *TKW* is considered to indicate the quality and composition of cereals.

**Figure 5:**
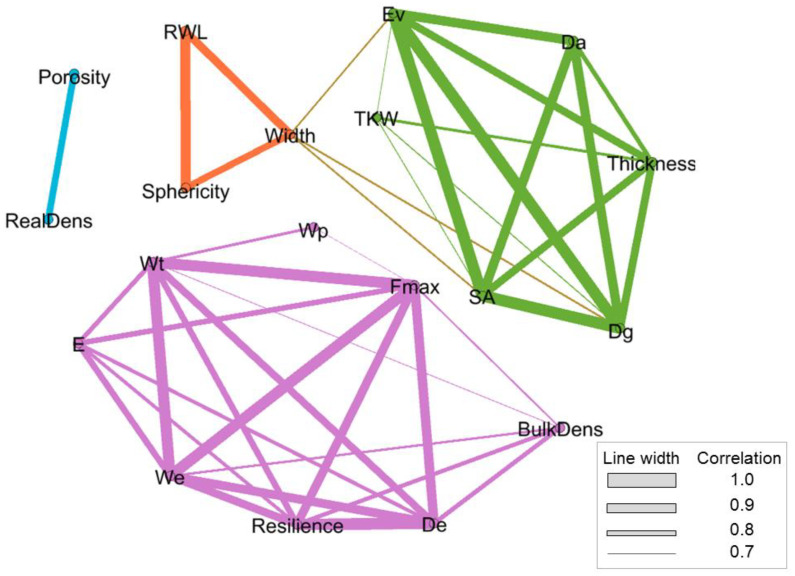
Correlation matrix among the evaluated parameters in three cereal types at two moisture levels (12% and 16%) demonstrating two main clusters with the higher relationship, where mechanical and viscoelastic properties stand out. The width bars represent the approximate *p*-value.

Figure 5 shows a 2D visual synthesis of the correlation analysis results. The correlations among variables are represented by four networks. The most extensive network (purple) groups the viscoelastic properties, which showed a strong correlation with *F_max_*. The viscoelastic properties also showed a strong correlation with $\rho_{b}$, which may be attributed to the moisture content. The next network (green) groups selected variables representing the biophysical, volume, and density properties with the highest correlation. This network indicates significant correlations among *D_g_*, *D_a_*, *E_v_*, *SA*, and *T*, and it is linked by *W* to a small network (orange) indicating the correlation between *R_WL_* and Φ. However, this small network showed no correlation with the purple network of viscoelastic properties. The final network (blue) is the smallest and groups density variables such as $\varepsilon_{v}$ and $\rho_{t}$.

Figure 4 indicates that the viscoelastic properties did not have a significant correlation with the appearance, geometric, and weight/volume/density properties of the kernels. In addition, *L*, *IKW*, and *H* did not have high correlation values (*p* > 0.7) and thus do not adequately explain the viscoelastic performance of biophysical properties of the kernels. Figure 5 suggests a general approach of arranging these variables in 2D space for PCA, which was performed to obtain the correlations among the four principal components with the highest significance. When an eigenvalue is greater than 1, it is considered statistically significant (*p* < 0.05). In this case, the eigenvalues of PC1, PC2, PC3, and PC4 were 10.91, 4.01, 3.17, and 1.63, respectively. Thus, they accounted for 85.77% of the variability in the observations.

**Figure 6:**
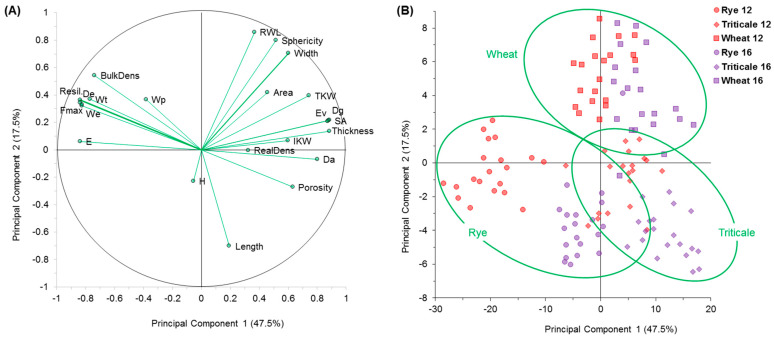
Principal component analysis (PCA) for the biophysical, geometric, mechanical, and viscoelastic properties of the cereal types at different moisture contents (12% and 16%). (**A**) Correlation matrix among PC1, PC2, and the evaluated variables. (**B**) Distribution of observations in the plane corresponding to PC1 and PC2 showing the distinction among cereals and moisture contents.

**Figure 7:**
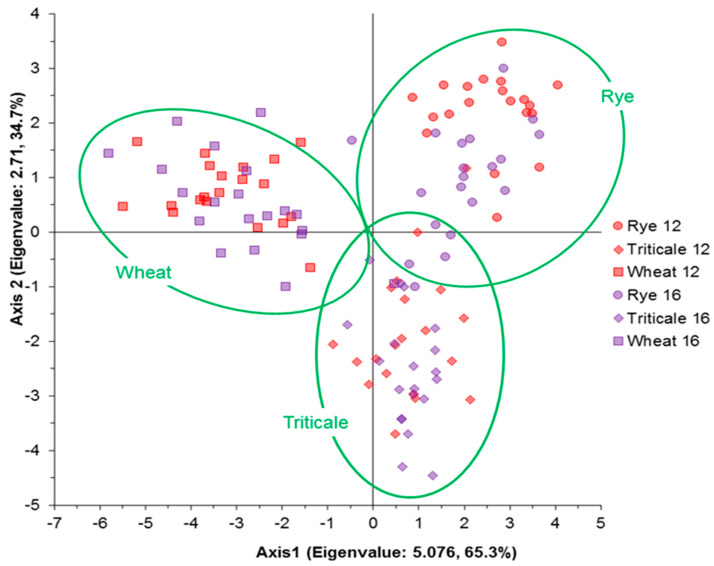
Discriminant analysis grouping different cereal types at two moisture contents (12% and 16%).

Figure 6 shows how the evaluated variables can influence kernel selection by cereal type and moisture content. In general, the viscoelastic properties strongly influenced rye selection at a moisture content of 12%. The weight, volume, and appearance properties influenced wheat selection at both moisture contents. Finally, the volume, density, and appearance properties influenced triticale selection. Figure 6A shows the correlation matrix between PC1 and PC2 and the evaluated variables. These two synthetic variables accounted for 64.91% of the variability in the observations. The viscoelastic properties showed a strong correlation with PC1 in opposition to the biophysical properties. The top left quadrant shows strong correlations for *F_max_*, *D_e_*, *W_t_*, *W_e_*, and *R*; this indicates that these variables express the tendency of the kernels or are redundant. *F_max_* may act as a broader reference for the other viscoelastic properties (Figure 5). The top right quadrant shows that *D_a_*, *D_g_*, *T*, *E_v_*, and *SA* expressed a similar behavior. For PC2, which was orthogonal to PC1, some biophysical properties such as *R_WL_*, *sphericity*, and *W* were opposed to *L*. Note that *H* did not have a significant correlation with PC3. Figure 6B depicts the distribution of observations in the plane corresponding to PC1 and PC2. The ellipses (green) represent significance at a 95% confidence level for each cereal type. PC1 and PC2 allowed a clear distinction between cereals and moisture levels. At 12% moisture content, kernels showed higher values for the viscoelastic properties; rye had the highest values, followed by wheat. At 16% moisture content, the kernels presented lower values, particularly for triticale (i.e., the kernels softened). In contrast, the biophysical properties showed higher values at the higher moisture content. For PC2, wheat kernels at both moisture contents showed the highest values for *R_WL_*, *sphericity*, and *W.* In contrast, rye presented higher values for *H*. Figure 7 shows the results of a discriminant analysis where variables such as $\rho_{t}$, $\rho_{b}$, $\varepsilon_{v}$, and *TKW* were eliminated. PC1 and PC2 could be used to correctly classify 96.67% of the observations according to cereal type. However, only 87.5% of the observations were correctly classified according to moisture content. This indicates that the uniaxial compression test and multivariate analysis including PCA are suitable for studying the differences among cereal types with different moisture contents.

## 4. Discussion

### 4.1. Biophysical Properties

Quality is a general term that can differ in meaning depending on the technical activity. From the viewpoint of cereal breeders with the goal of increasing the agronomical yield, quality is generally related to kernel appearance (*L*, *W*, *T,* and *R_WL_*), kernel weight (*TKW*), and test weight ($\rho_{b}$). The moisture content affects almost all appearance properties, as shown in Figure 1. Conditioning generally increases the values of these properties owing to water diffusion and the natural respiration process of these kernels [29]. Aprodu and Banu [7] noted that rye and triticale kernels have a higher water-absorption capacity than wheat. In this study, the results indicated that *ρ_b_* should be incorporated into kernel selection rather than sieving owing to the natural size variability of kernels. However, three triticale lines under different environmental conditions demonstrated significantly higher *SA* and *TKW* values than those reported by Dennett and Trethowan [6].

The geometric properties (*D_g_, D_a_, SA,* and *φ*) are essential for millers and determine the density of moving kernels during transportation [5]. In the present study, the rye kernels showed different geometric properties than wheat and triticale. This may be attributed to genomic characteristics because triticale is a hybrid cereal developed by combining the A and B genomes of wheat and the R genome of rye [30]. The differences in kernel properties and water adsorption can be attributed to the chemical composition and complex structure. Even for cereals that are botanically similar, the conformation of different tissues and cells, size, and anatomy change the spatial arrangement of the formed channels, which affects $\varepsilon_{v}$ and, in turn, the amount of water flow [29]. For the three cereal types, $\rho_{b}$ showed values that are suitable for international classification systems. For cereal breeders in developing countries, $\rho_{b}$ is helpful because they favor agronomical yield over quality. In addition, exporter countries give a special bonus for higher protein content, which is related to this parameter. For triticale kernels, *SA* and *TKW* differed from those for the other two cereal types. Those differences may be explained by the results of Dennett and Trethowan [6] who demonstrated that triticale kernels have a larger *SA*, which affects the ideal moisture content for improving the final milling yield and protein content.

The differences in geometric, volume, and density properties of the cereal types affected their behavior in response to the moisture content. *sphericity*, *ε_v_*, and *E_v_* were particularly affected by the moisture content. These results have tremendous importance because the kernel quality is related to specific parameters from the viewpoint of millers. During kernel handling, managing stresses is critical to calculating the effects of moisture and column kernel weight (load) on kernel deformation and breakability in silo during storage [31]. Agricultural products are generally damaged by static or dynamic external forces that affect the milling performance and, more rarely, by internal forces. During harvesting, transportation, and various handling and treatment processes, moving products against stationary or moving mechanical equipment affects the kernel quality. During milling operations, the volume, especially the dimensional properties of the kernel, determine the flour yield. Such properties affect kernel selection by sieve separators and the estimated extraction rate during size reduction processes. The axial dimensions of kernels are essential for calculating the kernel volume for modeling drying, aeration, heating, and cooling [10]. The results of this study showed that the axial dimensions of the three cereal types corresponded to optimal values reported in other studies, which can be used to produce specific food products.

The changes in *ε_v_* of the kernels can be associated with their chemical composition. During agronomic practices, kernels are frequently damaged by impacts [32]. *ε_v_* is related to the density of the endosperm, which is the principal constituent of a kernel, and its structure and morphology depend on the cereal type and agronomic practices [7]. Our results indicated that the biophysical properties of kernels are directly related to their chemical composition and are directly affected by their moisture content. Ensuring the quality of kernels before their transportation, distribution, storage, and processing is fundamental to reducing losses. Thus, developing basic assessments that can predict some end use features is needed. For example, a device that can evaluate these properties of intact kernels in the field or before/after harvesting could help breeders, producers, and the cereal industry determine the final use of various cereal types.

### 4.2. Mechanical and Viscoelastic Properties

The mechanical properties of kernels are a critical consideration for the design of processing equipment and storage facilities to reduce physical damage to the kernels and to reduce moisture uptake for avoiding fungus contamination [33]. The evaluation of the mechanical and viscoelastic properties of the cereals indicated significant differences in *H* and *F_max_*. This was expected because of the physiological differences among the cereal types. However, further analysis is needed to evaluate their milling behaviors. The PCA and discriminant analysis results indicated remarkable differences in the mechanical and viscoelastic properties depending on the cereal type and moisture content. Monitoring and predicting changes in the viscoelastic properties of kernels such as the water absorption capacity are valuable for technological purposes. Rye showed a higher *F_max_* and *H* than wheat and triticale. This can be explained by differences in the crease of the kernel, where the endosperm is firmly adhered to the aleurone. This inhibits efficient water hydration and uniform conditioning [7]. Thus, the role of the endosperm cell wall could be considered the molecular basis of *H* and other rheological features of the kernel [34].

Increasing the moisture content significantly affected the viscoelastic properties, particularly *W_t_*. Shpolyanskaya [35] considered *E* of greater practical significance for milling operations because it reflects the mechanical properties of the kernel in the form that it arrives at the rollers of the first break of a milling system [17]. Verjražka [36] observed that rye kernels had a high *E* and were difficult to mill; the flour yield from three rye varieties (two standard and one tuft rye) was lower than that of other cereals. Gómez et al. [4] demonstrated that the starch structure, enzyme content (amylases), and other soluble components of the pericarp have important effects on the rheological properties of rye dough during the bread-making process. In previous studies, some mechanical and viscoelastic properties of kernels showed a significant correlation with the dough performance and quality of bakery products [10,37]. The bread-making process is very complex, and it involves mixing, sheeting, fermentation, and baking in the oven. Selecting hard kernels is vital for obtaining good water absorption and a high bread loaf volume. Thus, *H*, *E*, *W_t_*, and other properties of the kernel (Figure 2) are fundamental for predicting the flour quality. To accommodate the interests of cereal breeders, millers, and bakers, PCA can be used to evaluate biophysical properties for their importance and to eliminate properties that are expensive and time-consuming to test. In this study, multivariate analysis was performed to eliminate properties with a minimal effect on the general quality of the evaluated cereals.

### 4.3. Multivariate Analysis

The multivariate analysis verified the degree of association among the evaluated properties and the similarity among cereal types. This analysis method can be used to reduce the number of variables to measure and to select variables with statistical utility. In addition, the obtaining of synthetic variables that are not correlated with each other (i.e., linear combinations of the initial variables) will allow their use in modeling methods such as linear regression or discriminant analysis. This is helpful for visualizing observations in a two-dimensional space to identify groups with high similarity [38]. The results of the discriminant analysis (Figure 7) indicate that future studies could focus on simplifying data acquisition to variables with the most significant influence. For example, the viscoelastic properties *F_max_*, *W_t_*, *W_e_*, and *E* could be considered for appearance properties such as *T* and geometric properties. *D_a_*, *D_g_,* and *E_v_* were the most important properties to measure. Future research will require discriminating variables with no significant effects on reducing the evaluation times and including some physicochemical properties for correlation with other variables that affect the cereal processing industry. Moreover, the optimal moisture content for conditioning should be further investigated because the composition properties differ among cereal types.

### 4.4. Limitations

This study considered the biophysical and mechanical properties of intact kernels and applied a validated methodology to measure the viscoelasticity. Multivariate analysis was applied to construct an exploratory view, which should be further complemented and validated with other advanced technologies such as image and thermal analyses for the development of devices/techniques that can predict the processing performance and/or final product traits. In addition, more cereal types should be considered to evaluate their performance at different moisture contents and their correlation with end-use characteristics. However, more research including diverse and numerous genotypes is required to confirm these preliminary findings. In this sense, this manuscript was more focused on performing a different way of data analysis since our previous works only applied ANOVA and Pearson correlations. The primary purpose is to analyze this data using PCA, from a fundamentally scientific point of view, in order to first, see if the method is suitable to differentiate the grain types and, secondly, to probe if it is worthy of evaluating in further research the end-quality parameters of products obtained from triticale and rye. Nevertheless, we are aware that complete research, including data from end-product quality, is still needed. 

## 5. Conclusions

This is the first study to use multivariate analysis for the validation of the uniaxial compression method developed to classify intact kernels by type and moisture content. A texture analyzer is a valuable equipment that is capable of measuring complex properties for evaluating kernel quality. The properties evaluated in this study allowed for appropriate and logical discrimination of kernels by cereal type and moisture content using PCA. Future studies will involve simplifying the data acquisition to consider only parameters that strongly influence the variability of the observations. For example, *F_max_* could be considered to predict the viscoelastic properties of kernels, and *D_a_*, *D_g_*, *T*, *E_υ_*, and *SA* could all express the same property. However, further mathematical modeling and image analyses are needed. The characterization of individual kernels by multivariate analysis and the application of a non-destructive test are a promising approach to indirectly measuring kernel properties associated with the quality of cereals and their products. The results of this study indicate that cereal kernels can be differentiated by quality and moisture content. This last feature is very important for classifying kernels before storage and predicting the behavior of conditioned kernels before milling, which would be useful for the manufacture of different cereal-based products.

## Data Availability

The datasets generated during and/or analyzed during the current study are available from the corresponding author upon reasonable request.

## Supplementary Materials

The supporting information can be downloaded at: https://www.mdpi.com/article/10.3390/foods12040808/s1.
